# Peripartum Cesarean Hysterectomy for Placenta Percreta: A Retrospective Analysis of Cases With and Without Extrauterine Disease

**DOI:** 10.7759/cureus.71169

**Published:** 2024-10-09

**Authors:** Khaled S Ismael, Mohamed S Abdelhafez, Alhussein A Mohamed, Mahmoud M Awad

**Affiliations:** 1 Department of Obstetrics and Gynecology, Mansoura University, Mansoura, EGY

**Keywords:** cesarean hysterectomy, pas, placenta accreta, placenta percreta, placenta previa

## Abstract

Objective

The aim of this study was to compare maternal morbidity and prenatal characteristics in placenta percreta cases with extrauterine disease and those with disease confined to the uterus.

Methods

A retrospective analysis of prospectively collected data was done at a large tertiary referral center in Egypt from October 2018 through February 2023. A total number of 73 cases who underwent primary cesarean hysterectomy with intrapartum confirmation of placenta previa percreta where there was evidence of at least placental invasion through uterine serosa (International Federation of Gynecology and Obstetrics (FIGO) grade 3) were included. Women without extrauterine disease (FIGO grade 3a) were compared to women with extrauterine disease where there was evidence of invasion of the bladder, parametrium, or both (FIGO grades 3b and 3c).

Results

Of 73 women included in the study, 46 women showed no evidence of extrauterine disease (group A), while the remaining 27 women had extrauterine disease (group B). The estimated amount of blood loss (mL) was 1,972 ± 671 in group A compared with 3,544 ± 899 in group B. There were no cases requiring massive transfusion in group A compared with eight of 27 cases (29.6%) in group B. Only two cases (4.3%) in group A had bladder injuries compared with 12 cases (44.4%) in group B. All 27 cases in group B required additional measures to control ongoing pelvic bleeding after hysterectomy compared with none of the cases in group A. The operating time (minutes) was 184 ± 30 in group A compared with 254 ± 42 in group B. Only five cases (10.9 %) in group A required postoperative intensive care unit admission, compared with 13 cases (48.1%) in group B.

Conclusion

Placenta accreta spectrum (PAS) with extrauterine disease represents the ultimate level of PAS surgery as it is more difficult, complex, and associated with the highest maternal morbidity. Highly specialized PAS centers are advised to categorize the disease into two different entities: PAS with extrauterine disease and PAS with disease confined to the uterus, as this may aid in improving the diagnostic accuracy of the severest PAS cases.

## Introduction

Placenta accreta spectrum (PAS) is considered one of the most dangerous pregnancy complications associated with maternal morbidity and even mortality. Massive bleeding can lead to multisystem organ failure and disseminated intravascular coagulation (DIC). Also, it is a common cause of intensive care unit (ICU) admission and peripartum hysterectomy [1-4]. The potential for bleeding correlates with the depth of villous invasion in the myometrium, the size of the affected area, and the presence or absence of invasion of extrauterine tissues [5].

The severity of the disease could be determined at the time of laparotomy using the International Federation of Gynecology and Obstetrics (FIGO) grading system [6], which generally classified PAS into the following: 1) grade 1 (G1), abnormally adherent placenta (placenta adherenta or creta); 2) grade 2 (G2), abnormally invasive placenta (increta); and 3) grade 3 (G3), abnormally invasive placenta (percreta), which was further divided into a) grade 3a (G3a), limited to the uterine serosa; grade 3b (G3b), with urinary bladder invasion; and c) grade 3c (G3c), with invasion of other pelvic tissue/organs.

Uterine preservation surgery for PAS has become increasingly common over the past two decades and could be applied for less severe forms of PAS [7]. This could be achieved by resection of the focally embedded placenta and uterine repair [8]. As a result of the increased incidence of PAS [9], most obstetricians became familiar with the management of mild forms of the disease. For women with severe PAS, especially those with confirmation of placenta percreta at the time of surgery, cesarean hysterectomy remains the definitive measure [10]. Several techniques for peripartum hysterectomy for PAS have been described in the literature [11-14].

Four factors contribute to the difficulty and complexity of surgery for severe PAS. First, the placental bulk lies in the narrowest part of the pelvis, making identification of vital structures (such as distal ureters and uterine arteries) from their origin difficult [15]. Second, there is a presence of invasion of the placenta into the bladder, broad ligament, or parametrium. Third, abnormal neovascularization is present. Fourth, there is difficulty in identifying anatomical planes, particularly the correct uterovesical interface from previous cesarean delivery (CD) and associated fibrosis [16].

Even with experienced hands, surgery for placenta previa percreta with extrauterine invasion still carries the risk of massive bleeding from vessels arising outside the territories of the internal iliac arteries, which represents an additional blood supply that mostly comes from external iliac arteries [15].

Identifying a subgroup of PAS with extrauterine disease as a separate category and distinguishing it from less severe forms became essential. Improving diagnostic accuracy for better prenatal prediction, referral to most specialized PAS centers, and finally implementing adjunct measures that contribute to hemostasis and reduce the burden of surgical procedures in such complex cases should be the main concern in the future. Because most publications in PAS were concerned with studying the disease as a single unit, we aimed in this study to compare maternal morbidity and prenatal characteristics in placenta percreta cases with extrauterine disease and those confined to the uterus.

## Materials and methods

Study design

This was a retrospective analysis of prospectively collected data from women who underwent primary cesarean hysterectomy for placenta previa percreta at Mansoura University Hospital (MUH), Egypt, between October 2018 and February 2023. This study was approved by the Mansoura Faculty of Medicine Institutional Research Board (code number R.23.06.2211) and registered with ClinicalTrials.gov, identifier NCT05979181.

The main inclusion criterion was women who underwent primary cesarean hysterectomy for placenta previa percreta (FIGO grade 3), diagnosed by color flow Doppler and confirmed intraoperatively. Women without extrauterine disease (FIGO grade 3a) were compared with women with extrauterine disease (FIGO grades 3b and 3c). Women with any of the following criteria were excluded from the study: 1) medical conditions complicating pregnancy; 2) blood diseases or bleeding tendencies; 3) less severe PAS (FIGO grades 1 and 2 diseases); and 4) women who have undergone a uterine preservation surgery, such as resection of the invaded lower uterine segment (LUS) with the attached placenta.

Maternal demographic data, clinical characteristics, ultrasound findings, perioperative details, and maternal and neonatal outcomes were collected and recorded in a database by the same multidisciplinary team. All women were informed about the risks and complications of the operation. Written informed consent was obtained from each woman before performing any intervention.

Preparation and technique of cesarean hysterectomy

Blood products were prepared to be available for transfusion. The delivery was performed by a multidisciplinary team, including an experienced obstetrician, an assistant, an experienced anesthesiologist, and a pediatrician. All participants received general anesthesia.

The choice of abdominal incision was decided on a case-by-case basis. A midline periumbilical incision was performed when the main bulk of the placenta was anterior and extending toward the level of the umbilicus or there was suspicion of lateral invasion by prenatal imaging. Otherwise, a modified transverse incision was performed.

A transverse incision was performed in the uterus above the upper border of the placenta or in the uterine fundus, followed by extraction of the fetus. The umbilical cord was clamped without attempting to detach the placenta. The uterus with the placenta inside was exteriorized.

**Figure 1:**
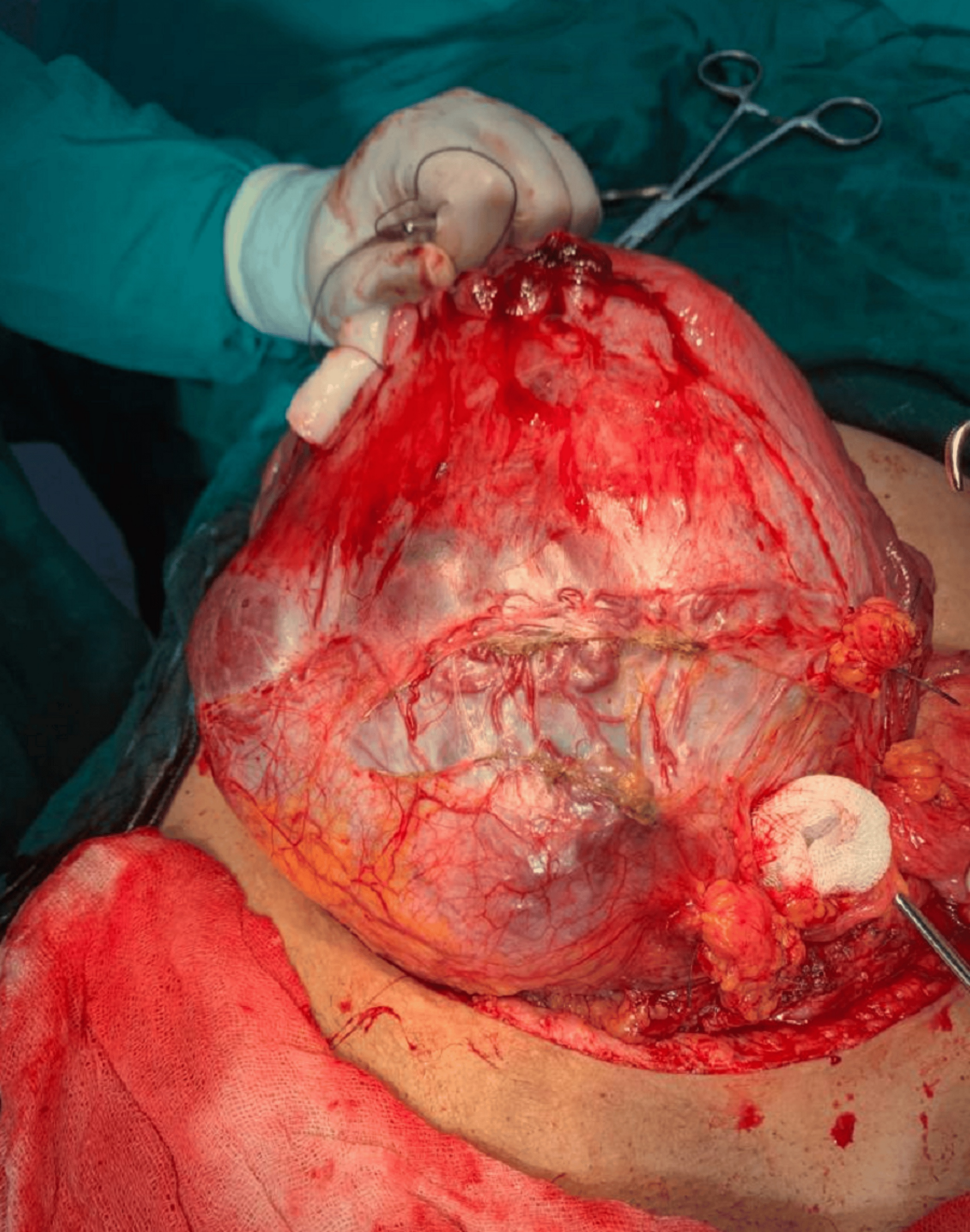
Intraoperative photo of PAS showing placental bulge, bluish coloration with evidence of abnormal vascularity (FIGO grade 3 PAS). FIGO, International Federation of Gynecology and Obstetrics; PAS, placenta accreta spectrum

A primary survey was done by inspecting the placental bed, and FIGO grade 3 was diagnosed based on the following criteria [6] (Figure 1): a) presence of bluish discoloration and distension of the LUS; b) presence of excessive hypervascularity running parallel craniocaudally in the uterine serosa; and c) placental tissue was seen to be invading through the uterine serosa.

The decision to perform a cesarean hysterectomy was made by the multidisciplinary team based on the severity of the placental invasion and the woman's desire to preserve her future fertility. If a hysterectomy was decided, the hysterotomy incision was closed. The round ligament was divided and ligated. The utero-ovarian ligament and utero-ovarian anastomotic vessels were clamped, divided, and ligated with absorbable suture. Retroperitoneal dissection was done by identifying and slinging the ureter attached to the medial peritoneal leaflet. The paravesical spaces were dissected. The peritoneum over the bladder was incised from one round ligament to the other. The bladder dome was carefully grasped with Babcock forceps (KLS Martin, Tuttlingen, Germany) and gently tractioned toward the symphysis pubis.

A secondary assessment of disease severity was performed. If the placenta was localized to the uterus with no invasion into any other organ, it was classified as FIGO grade 3a. If only the posterior bladder wall was invaded by the placenta, this was graded as FIGO grade 3b. If the placenta invaded the broad ligament and/or parametrium, this was graded as FIGO grade 3c.

**Figure 2:**
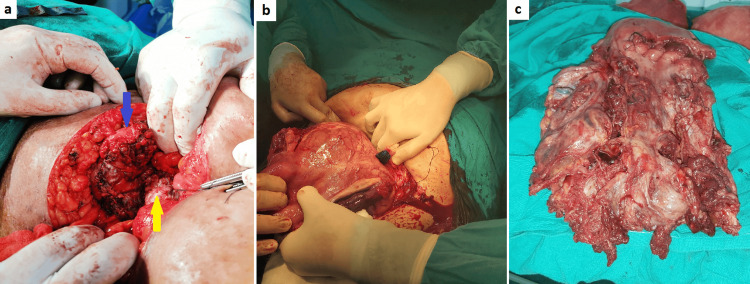
Placenta percreta with extrauterine disease. (a) Intraoperative photo for patient no. 57 after subtotal hysterectomy for PAS showing invasion of posterior wall of the bladder (FIGO grade 3b PAS), and there was a complex bladder injury that was repaired by a urologist (yellow arrow refers to cervical stump and blue arrow refers to the bladder after repair). (b) Intraoperative photo for patient no. 23 showing placenta percreta extending into the right broad ligament (FIGO grade 3c PAS). (c) Subtotal hysterectomy specimen of the same patient (patient no. 23). FIGO, International Federation of Gynecology and Obstetrics; PAS, placenta accreta spectrum

The further steps of the hysterectomy were determined by the surgical team with the following techniques, reserved for FIGO grades 3b and 3c (Figure 2): 1) ureterolysis, in which if there was a lateral invasion of the placenta, the ureter was grasped and separated from the highly vascular placental tissue, and ureteric tunneling was required for cases with evident parametric invasion; 2) uterine arteries were ligated at or near their origin from the internal iliac arteries; and 3) use of lateral approach to bladder dissection, in which the previously dissected paravesical space was used as a guide into the vesicouterine space in an area below the site of maximum invasion, followed by dissection of the invaded portion of the bladder under visualization with the attached portion of the placental tissue. Alternatively, the bladder was intentionally opened, and a part of the posterior wall above the ureteric orifices was resected with the attached placenta, followed by bladder repair by a urologist.

After amputation of the uterus, the case was observed for ongoing pelvic bleeding from extensive neovascularization or abnormal placental invasion. If present, we tried one or more of the following methods: 1) direct or distal vascular attack, in which the abnormal vessels were systematically grasped with DeBakey forceps (KLS Martin, Tuttlingen, Germany), and a mixture right angle forceps was applied below it followed by ligation; 2) the second was to perform sewing suture on the back of bladder and pelvic peritoneum; or 3) the third method was to do a proximal vascular control through ligating the anterior division of internal iliac artery or to ligate the superior vesical and uterine arteries from their origin if not previously ligated.

Blood loss measurement and replacement protocol

Blood loss was estimated using a combination of direct measurement and gravimetric methods [17], as previously described by Thabet et al. [18].

Outcome measures

The main outcome measures of this study were the following: 1) estimated amount of intraoperative blood loss; 2) the amount of blood products transfused, including packed red blood cells (PRBCs), fresh frozen plasma (FFP) and platelets; 3) use of a massive transfusion protocol (MTP), which was defined as transfusion of more than four units of packed RBCs within one hour with anticipated ongoing bleeding or transfusion of more than 10 units of packed RBCs within 24 hours [19]; 4) duration of operation; 5) admission to the ICU; and 6) postoperative hospital stays.

Statistical analysis

The SPSS version 20.0 (IBM SPSS Statistics for Windows, Armonk, NY) was used for statistical analysis. Continuous variables were presented as mean ± standard deviation (SD) or median (minimum-maximum) as appropriate. The Kolmogorov-Smirnov and Shapiro-Wilk tests were used to test the normality distribution of continuous variables. The Student t-test was used to compare the normally distributed continuous variables among the two studied groups, while the Mann-Whitney U test was used to compare continuous variables without normal distribution. Categorical variables were presented as frequencies and percentages, and they were compared by the chi-square test with Fischer's exact test as a correction for the chi-square test when >25% of cells have a count < 5. The p-values were considered statistically significant at level ≤0.05.

## Results

A total of 73 women who underwent primary cesarean hysterectomy for placenta previa percreta (FIGO grade 3) were included in the study. In 46 women (group A), there was no evidence of extrauterine disease (FIGO grade 3a), while in the remaining 27 women (group B), there was evidence of extrauterine disease (eight cases FIGO grade 3b and 19 cases FIGO grade 3c). Both groups were similar to each other in age, body mass index (BMI), gravidity, parity, and number of previous CD. Ultrasound results showed that subplacental hypervascularity and bladder wall interruption were significantly more prevalent in the extrauterine disease group (Table 1).

**Table 1:** Demographic and clinical characteristics of both groups * Expressed as mean ± SD, and p-value was calculated using the Mann-Whitney U test. † Expressed as median (minimum-maximum), and p-value was calculated using the Mann-Whitney U test. ‡ Expressed as frequency and percentage, and p-value was calculated using the chi-square test with Fischer's exact test as a correction for the chi-square test when >25% of cells have count < 5. BMI, body mass index; CD, cesarean delivery

		Group A (n = 46)	Group B (n = 27)	p-value
Age (years) *		33.56 ± 5.08	32.81 ± 4.49	0.631
BMI (kg/m^2^) *		29.64 ± 3.98	29.53 ± 3.71	0.973
Gravidity ^†^		4.5 (2-13)	5 (3-9)	0.665
Parity ^†^		3 (1-5)	3 (2-7)	0.650
Previous CD ^†^		3 (1-5)	3 (2-6)	0.496
Multifetal pregnancy ^‡^		2 (4.3%)	0 (0.0%)	0.527
Ultrasound findings ^‡^				
Lacunae	G1	3 (6.5%)	0 (0.0%)	0.126
	G2	27 (58.7%)	12 (44.4%)	
	G3	16 (34.8%)	15 (55.6%)	
Retroplacental hypoechoic line		0 (0.0%)	0 (0.0%)	1.000
Myometrial thickness < 1 mm		46 (100%)	27 (100.0%)	1.000
Bladder wall interruption		2 (4.3%)	15 (55.6%)	<0.001
Uterovesical hypervascularity		45 (97.8%)	27 (100.0%)	0.440
Subplacental hypervascularity		32 (69.6%)	26 (96.3%)	0.006

There was no significant difference in the gestational age at delivery between both groups. No cases of ureteric injury were recorded in either group, but the incidence of urinary bladder injury was significantly higher in cases with extrauterine disease (p < 0.001). The operative time, ICU admission, and postoperative hospital stay were significantly longer in cases with extrauterine disease. In addition, cases with extrauterine disease have a significantly lower postoperative hemoglobin level and a significantly higher hemoglobin deficit (Table 2).

**Table 2:** Operative and postoperative characteristics of both groups * Expressed as mean ± SD, and p-value was calculated using the Mann-Whitney U test. † Expressed as frequency and percentage, and p-value was calculated using the chi-square test with Fischer's exact test as a correction for the chi-square test when >25% of cells have count < 5. ‡ Expressed as median (minimum-maximum), and p-value was calculated using the Mann-Whitney U test. ICU, intensive care unit

	Group A (n = 46)	Group B (n = 27)	p-value
Preoperative hemoglobin level (g/dL) *	11.05 ± 0.82	10.93 ± 0.86	0.245
Gestational age at delivery (weeks) *	35.86 ± 2.82	35.22 ± 3.63	0.443
Ureteric injury ^†^	0 (0.0%)	0 (0.0%)	1.000
Urinary bladder injury ^†^	2 (4.3%)	12 (44.4%)	<0.001
Extensive ureterolysis ^†^	0 (0.0%)	19 (70.4%)	<0.001
Ligation of uterine artery at or near its origin ^†^	0 (0.0%)	19 (70.4%)	<0.001
Additional measures to control pelvic bleeding after hysterectomy ^†^	0 (0.0%)	27 (100.0%)	<0.001
Duration of operation (minute) *	184 ± 30	254 ± 42	<0.001
Postoperative hemoglobin level (g/dL) *	9.84 ± 1.29	8.24 ± 1.23	<0.001
Hemoglobin deficit (g/dL) *	1.21 ± 1.00	2.69 ± 1.05	<0.001
Relaparotomy ^†^	0 (0.0%)	1 (3.7%)	0.370
Admission to ICU ^†^	5 (10.9%)	13 (48.1%)	<0.001
Coagulopathy ^†^	0 (0.0%)	1 (3.7%)	0.370
Postoperative hospital stay (days) ^‡^	7 (6-16)	14 (7-21)	<0.001

The estimated amount of blood loss was significantly higher in cases with extrauterine disease (1,972 ± 671 mL vs. 3,544 ± 899 mL; p < 0.001). In cases with extrauterine disease, a significant increase in transfusion amounts of crystalloids, colloids, and all blood products (PRBCs, FFP, and platelets) was observed. MTP was used in eight cases with extrauterine disease, and none of the cases with disease confined to the uterus. Furthermore, the need and amount of postoperative blood transfusions were significantly higher in cases with extrauterine disease (Table 3).

**Table 3:** Blood loss and fluid and blood product transfusion in both groups * Expressed as mean ± SD, and p-value was calculated using the Mann-Whitney U test. † Expressed as frequency and percentage, and p-value was calculated using the chi-square test with Fischer's exact test as a correction for the chi-square test when >25% of cells have count < 5. ‡ Expressed as median (minimum-maximum), and p-value was calculated using the Mann-Whitney U test. FFP, fresh frozen plasma; MTP, massive transfusion protocol; PRBCs, packed red blood cells

	Group A (n = 46)	Group B (n = 27)	p-value
Estimated amount of blood loss (mL) *	1,972 ± 671	3,544 ± 899	<0.001
Amount of crystalloids transfusion (mL) *	3,065 ± 533	4,019 ± 294	<0.001
Colloids transfusion			
Patients received transfusion ^†^	33 (71.7%)	27 (100.0%)	0.002
Amount transfused (mL) *	641 ± 443	1,000 ± 0	<0.001
PRBCs transfusion			
Patients received transfusion ^†^	46 (100.0%)	27 (100.0%)	1.000
Amount transfused (units) ^‡^	4 (1-9)	7 (5-10)	<0.001
FFP transfusion			
Patients received transfusion ^†^	43 (93.5%)	27 (100.0%)	0.175
Amount transfused (units) ^‡^	4 (0-9)	7 (5-10)	<0.001
Platelet transfusion			
Patients received transfusion ^†^	1 (2.2%)	18 (66.7%)	<0.001
Amount transfused (units) ^‡^	0 (0-5)	5 (0-10)	<0.001
Use of MTP ^†^	0 (0.0%)	8 (29.6%)	<0.001
Postoperative blood products transfusion			
Patients received transfusion ^†^	15 (32.6%)	24 (88.9%)	<0.001
Amount transfused (units) ^‡^	0 (0-3)	2 (0-3)	<0.001

## Discussion

Principal findings

The main finding of our study is that PAS cases with extrauterine disease had higher morbidity than those with disease confined to the uterus. This was manifested by higher blood loss, more transfusions of blood products, higher incidence of urological injuries, higher ICU admission rate, and higher postoperative hospital stay.

In addition, surgery for PAS with extrauterine disease was more complex, difficult, and prolonged. This could be explained by the need for additional maneuvers in the form of extensive ureterolysis, ligation of the uterine artery at or near its origin, and difficult bladder dissection with a higher incidence of complex bladder injuries. When PAS was confined to the uterus, most bleeding was controlled after amputation of the uterus or even at the time of bilateral clamping of the uterine pedicles. In contrast, in PAS with extrauterine disease, ongoing pelvic bleeding was present after hysterectomy, and additional techniques were required to achieve hemostasis.

**Figure 3:**
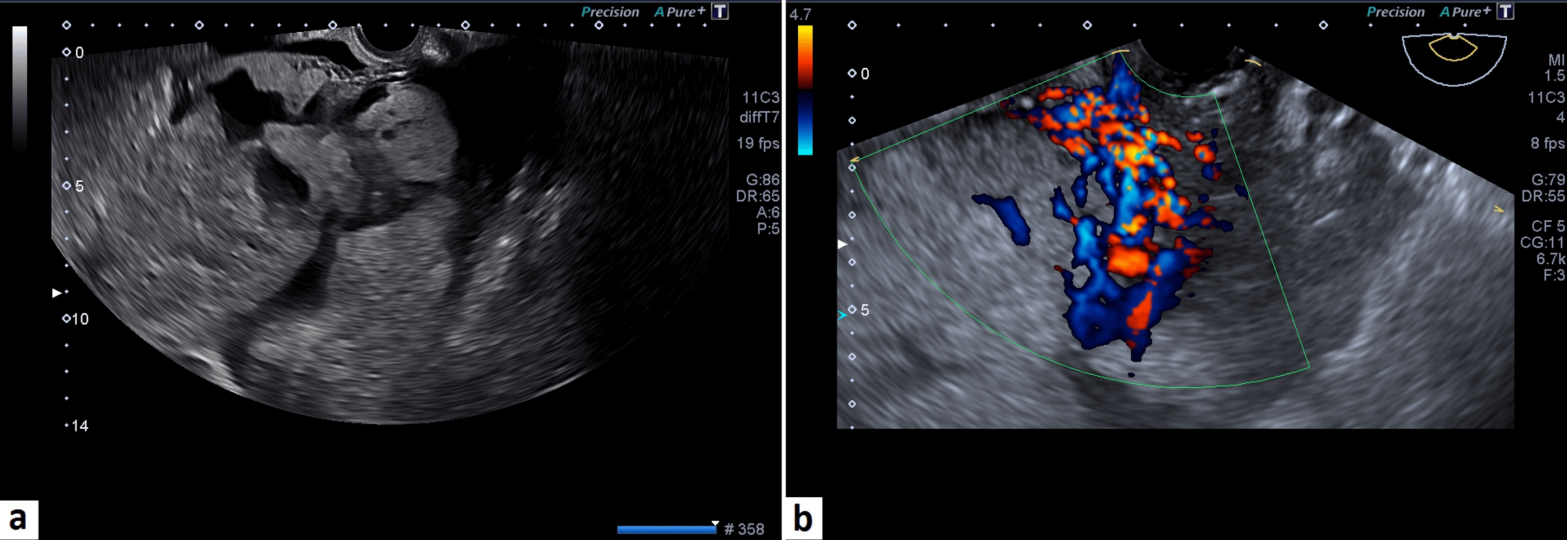
(a) Ultrasound image of PAS showing multiple placental lacunae and interruption of uterovesical interface. (b) Color Doppler image of PAS showing subplacental hypervascularity (in the inferior part of lower uterine segment). PAS, placenta accreta spectrum

In our study using 2D grayscale ultrasound and 2D color Doppler, we found that the presence of bladder wall interruption and subplacental hypervascularity in the inferior part of the LUS was significantly more common in the group with extrauterine disease (Figure 3).

Results in the context of what is known

Soleymani Majd and his colleagues [16] recently described a modified surgical technique for severe cases of placenta percreta, including only FIGO grades 3b and 3c PAS cases. They found that this technique led to a significant improvement in patient outcomes. Our surgical technique had the same principles described in their study. The main difference was that we preferred longitudinal incision for suspected severe PAS. The reason for this is that we have encountered cases where the uterus adhered to the anterior abdominal wall from prior repeated CDs, which inevitably led to the opening of the anterior uterine wall with the underlying placenta and catastrophic bleeding during abdominal entry. The longitudinal incision allows extension above the umbilicus and safe entry.

A retrospective study by Marcellin and his colleagues found that severe maternal morbidity was much more frequent in women with placenta percreta than those with placenta accreta despite multidisciplinary planning. This study recommended leaving the placenta in situ as a safe approach for severe PAS [20].

In contrast to our study, which had focused mainly on the surgical role alone in the treatment of severe PAS, other studies addressed the role of interventional radiology in these cases. The use of prophylactic resuscitative endovascular balloon occlusion of the aorta (REBOA) during cesarean hysterectomy for PAS has been shown to be associated with a significant reduction in blood loss during surgery [21].

Another method aims at a more distal arterial occlusion called the PASTIME (Placenta Accreta Spectrum Treatment with Intraoperative Multivessel Embolization) protocol. The use of this method was associated with a reduction of blood loss and transfusion requirements in cases with PAS [22]. Unfortunately, these techniques are not available at our institute, but we hope to integrate them as a crucial part of the treatment of cases with severe PAS soon.

We compared our prenatal ultrasound findings with the study by Cali and colleagues [23] that aimed to establish a prenatal ultrasound staging system for PAS and concluded that signs associated with severe disease included uterovesical hypervascularity (PAS2) and evidence of increased vascularity in the lower part of LUS with the possibility of extension into the parametrial region (PAS3) when combined with at least one additional PAS sign. Our results agreed with the results of this study.

Strengths and limitations

The main strength of our study is that, to the best of our knowledge, this is the first study to compare cases of placenta percreta with extrauterine disease with cases without extrauterine disease. The inclusion of a relatively large number of cases (73 cases of cesarean hysterectomy for severe PAS) is another strength.

A limitation of our study is that we did not refer to the histopathological examination of hysterectomy specimens. Although histopathological examination was routinely performed in all cases, we preferred not to include it in the study for the following reasons: 1) this pathological examination has no impact on a patient’s outcome or prognosis as it does not aid in decision-making at the operating table; therefore, we relied on prenatal diagnosis by imaging and the intraoperative grading system to determine disease severity and therefore our treatment plan; and 2) to prove whether extrauterine disease is due to actual villous invasion or just neovascularization is difficult. This is because the treatment of PAS aims at ensuring hemostasis, rather than radical treatment as that done for cancer, which means that the specimen sent for pathological examination may not be representative of the entire spectrum of the disease with the possibility of under-diagnosis. Another critical point is that regardless of the nature of extrauterine disease, it is associated with increased patient morbidity and the risk of severe bleeding. Therefore, finding solutions to deal with this serious condition is more important than proving a theoretical point; 3) histologic examination is subjected to multiple biases and is highly dependent on the experience of the operator.

## Conclusions

We can conclude that PAS with extrauterine disease represents the ultimate level of PAS surgery as it is more difficult, complex, and associated with the highest patient morbidity. Prenatal ultrasound with the presence of interruption of uterovesical interface combined with uterovesical hypervascularity or subplacental hypervascularity in the inferior pole of the uterus may be suggestive of most severe cases with PAS. Highly specialized PAS centers are advised to categorize the disease into two different entities: PAS with extrauterine disease and PAS with disease confined to the uterus, as this may aid in improving the diagnostic accuracy of the severest PAS cases.
